# Successful Repair of a Blunt Injury to the Donor's Pulmonary Artery and Subsequent Use of the Lungs

**DOI:** 10.1016/j.atssr.2026.02.014

**Published:** 2026-02-28

**Authors:** Eugene Golts, Samvel Gaboyan, Alejandro Bribriesco, Travis Pollema, Ian Glenn, Rebecca Golts, Aideen Afshar, Aiden Walters, Kamyar Afshar

**Affiliations:** 1 Division of Cardiovascular and Thoracic Surgery, Department of Surgery, University of California, San Diego, La Jolla, California; 2 Division of Pulmonary, Critical Care, and Sleep Medicine & Physiology, Department of Medicine, University of California, San Diego, La Jolla, California; 3 Case Western Reserve University School of Medicine, Cleveland, Ohio; 4 Section of Cardiothoracic Surgery, Surgical Services, Louis Stokes Cleveland Veterans Affairs Medical Center, Cleveland, Ohio; 5 University of California, San Diego, Health, La Jolla, California

## Abstract

A significant proportion of lungs accepted for lung transplantation are not transplanted due to chest trauma. We present a successful repair of a blunt injury to the pulmonary artery of the donor during lung transplantation. Lungs with this type of injury can be transplanted safely after repair, potentially expanding the donor pool without compromising the postoperative course.

Although a quarter of the lung donor pool comprises cases involving blunt trauma, pulmonary artery injuries due to blunt trauma are uncommon and often fatal.^1^^,^^2^ Prior studies have shown that thoracic trauma is associated with increased odds of lungs being declined in the donor operating room before procurement; however, data on the incidence of blunt injury to the pulmonary artery is limited.^3^^,^^4^ Herein, we present the case of a patient who underwent successful lung transplantation after the repair of a blunt injury to the pulmonary artery of the donor.

A 21-year-old man was involved in a high-speed motorcycle accident. On the scene, when attempting to intubate with the primary survey, blood was noted in the oropharynx. The patient suffered cardiac arrest when placed on a cardiac monitor. He was successfully resuscitated in the field and admitted to a level 1 trauma unit. On secondary survey, he had extensive head and chest injuries. Specific chest injuries included a descending thoracic aortic pseudoaneurysm, measuring 1.7 × 0.8 cm, with no associated dissection on computed tomography, along with small bilateral pleural effusions, complete atelectasis of the left lower and right lower lobes, and multiple thoracic spine fractures (Figure 1). The patient's neurological condition rapidly deteriorated. Through discussion with family and the organ procurement organization, he became an organ donor.

**Figure 1:**
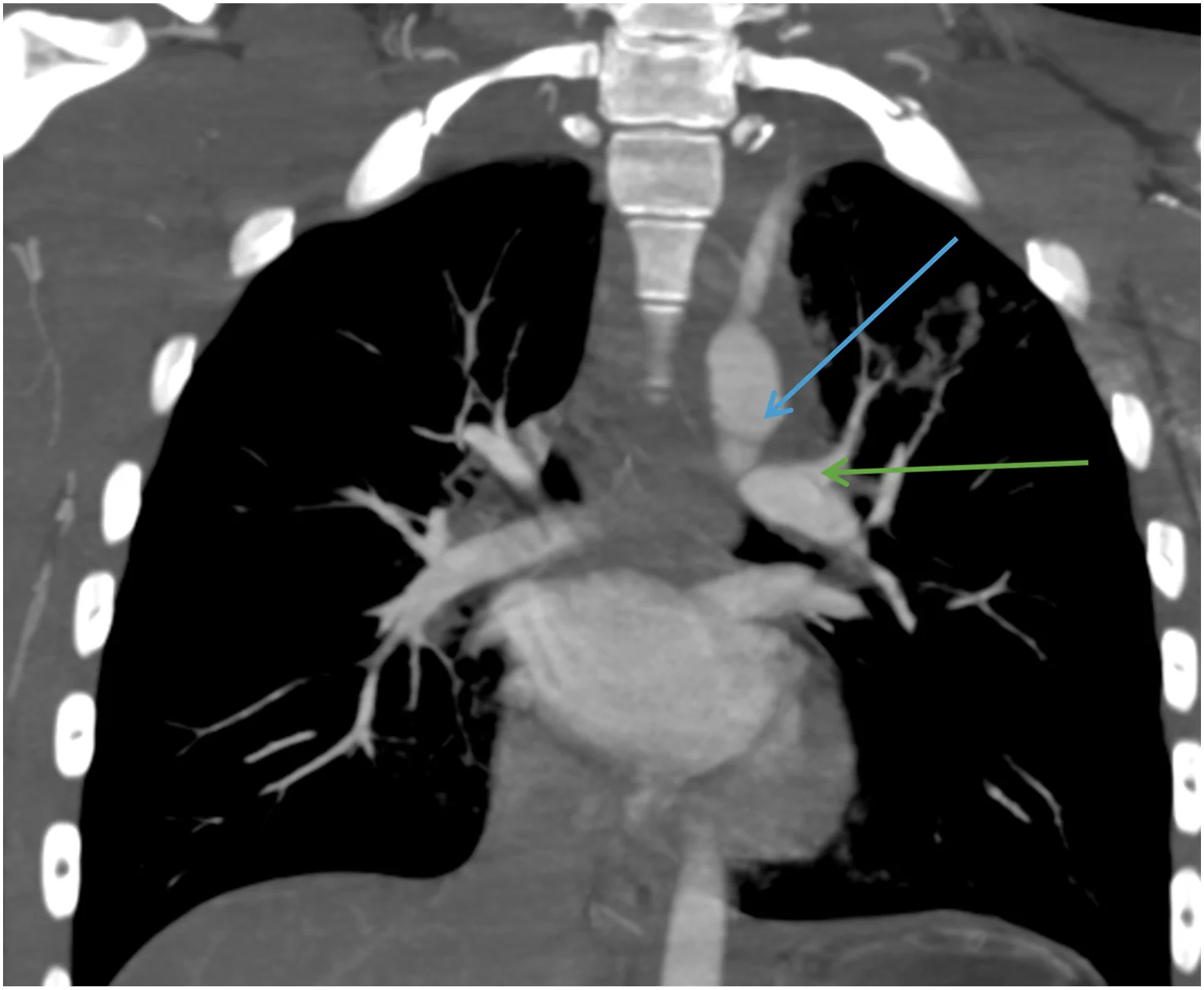
Coronal view chest computed tomography of the donor with the blue arrow pointing to the aortic injury (so called, “distracting injury”) and the green arrow pointing to the left pulmonary artery injury (“missed injury”). The imaging demonstrated an aortic injury in the vicinity of the ligamentum arteriosum.

His lungs were evaluated and accepted for transplantation for a 50-year-old woman with severely obstructive idiopathic bronchiectasis complicated by *Mycobacterium abscessus* and *Aspergillus niger* infections and acute-on-chronic respiratory failure with hypoxemia and hypercapnia.

Organ procurement proceeded without complications, with both antegrade and retrograde flushes performed in situ using PERFADEX (XVIVO Perfusion AB). During procurement, a previously identified injury to the aortic isthmus was noted, along with an associated hematoma in the general area of the ligamentum arteriosum and blood staining of the adipose and lymphatic tissue in the area. The lungs were preserved and transported using the Paragonix BAROguard system.

The recipient operation commenced in the fashion adopted at our center. Briefly, surgery was performed through a transverse bilateral thoracosternotomy on venoarterial extracorporeal membrane oxygenation with central cannulation. Bilateral hilar dissection was performed. Recipient pneumonectomy on the right side was performed by transecting the vascular structures with Endo GIA stapler (United States Surgical Corp) vascular loads. The bronchus was transected sharply.

Upon delivery to our transplant center and after the lungs were separated on the back table, surprisingly, transmural injury to the left pulmonary artery just distal to the ligamentum arteriosum attachment was identified (Figure 2A). The tear was oriented transversely, perpendicular to the longitudinal axis of the pulmonary artery in that area and measured ∼1.5 cm in length. It appeared to emanate from the intraluminal surface of the artery as the overlying parenchyma of the left lung appeared intact. The remainder of the donor lung examination was satisfactory.

**Figure 2:**
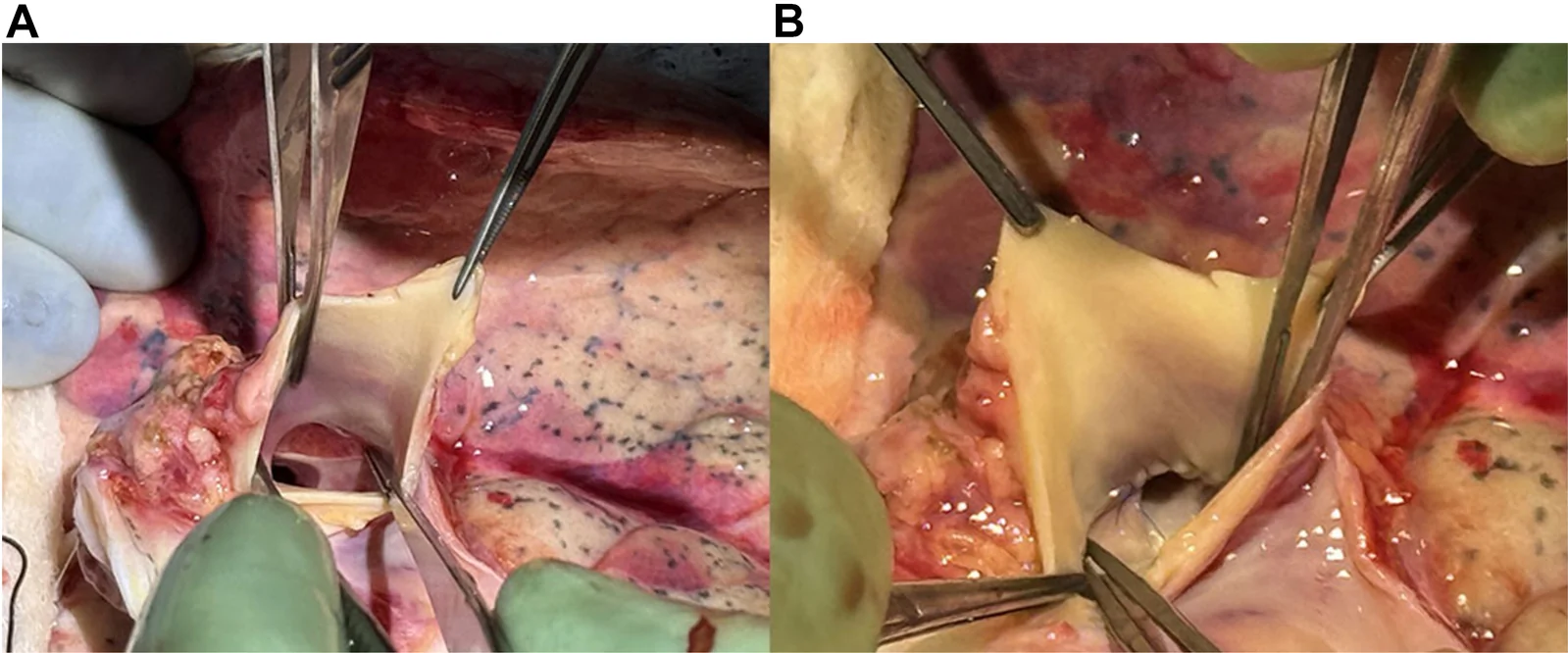
(A) Visualization of the injury in the left pulmonary artery. Note that the injury is transmural, as evidenced by the lung parenchyma visible. (B) Repair of the injury using Prolene suture in 2 layers.

The decision was made to proceed with the implantation. The donor's lungs were prepared on the back table with the appropriate vascular and bronchial cuffs fashioned. The tear in the left pulmonary artery was repaired using a running 6-0 Prolene (Ethicon) suture in 2 layers (Figure 2B). We were prepared to repair the artery with the patch of the donor pericardium should our primary repair fail.

The donor's right lung was delivered into the recipient's operative field and implanted in the usual manner adopted at our institution. Briefly, the donor bronchus was anastomosed to the recipient bronchus in an end-to-end fashion with the running single-layer 4-0 Prolene suture, followed by the donor left atrial cuff anastomosis to the recipient atrial cuff with the 4-0 Prolene running suture, and finally following the pulmonary artery of the donor anastomosis to the recipient pulmonary artery with the running 5-0 Prolene suture. Left recipient pneumonectomy and left donor lung implantation were performed in a fashion identical to the one described for the right side.

After a brief reperfusion period, the patient was weaned off the extracorporeal membrane oxygenation and decannulated. Excellent graft function was confirmed with a Pao_2_-to-fraction of inspired oxygen ratio of 400 on 40% fraction of inspired oxygen and good compliance of the newly implanted lungs. The remainder of the operation and recovery were uneventful.

The patient recovered well without any primary graft dysfunction and was discharged on postoperative day 12. At 8 months posttransplant she was without any pulmonary complications.

## Comment

Pulmonary artery injuries due to blunt trauma are rare and frequently lethal.^2^ Therefore, there is paucity of data available on the incidence of this injury.^4^ This report describes a case of donor lungs with pulmonary artery blunt injury identified preimplantation, repaired, and used successfully for the lung transplant. As reported in the Organ Procurement and Transplant Network data set, blunt injury was identified as the mechanism of death in 24.5% of lung organ donors (12,625 of 51,613) between 1995 and 2024.^1^ Motor vehicle accident with presumed blunt injury as the circumstance and mechanism of death is present in up to 16.9% of (8740 of 51,613).^1^ In 2024, the percentage of lung donors involved in a motor vehicle accident was 12.6% (467 of 3697), among which blunt injury was the presumed mechanism of death for 94.2% (440 of 467).^1^

In these settings, evaluation and a high index of suspicion of the injury to the pulmonary artery are necessary. In our case, the injury was relatively distal to the usual site of the pulmonary artery anastomosis, highlighting the need for careful examination of all hilar structures for potential injury. If the injury is identified, ex vivo perfusion of the organ should only be considered after the injury is repaired or avoided altogether, and hypothermic static preservation is preferred.

In our practice, we use donor thoracic aorta for the reconstruction of various vascular structures in transplants when the need arises, and had we known about the tear, we would have considered harvesting additional donor thoracic aorta. We felt the remaining pulmonary artery length would not allow for a tension-free anastomosis, prompting us to proceed with direct repair. In addition, the tear was covered by lung parenchyma, and we felt that performing a direct anastomosis in that area would be prohibitively difficult and fraught with the risk of additional injury to the lung parenchyma.

Finally, we felt that performing single right lung implantation and relisting for the single left lung later was entertained but deemed not beneficial to the patient for 2 reasons. First, the donor was colonized with several drug-resistant organisms, and we felt that choice of single-lung transplant would subject her to a higher risk of complications. Second, we had already performed bilateral hilar dissection and dissected the lung from the chest wall and felt that recovery would be complicated with the multiple parenchymal injuries present.

Lungs with this type of injury can be used safely after repair, potentially expanding the donor pool without compromising the postoperative course.
